# A retrospective research on non-suicidal self-injurious behaviors among young patients diagnosed with mood disorders

**DOI:** 10.3389/fpsyt.2022.895892

**Published:** 2022-07-22

**Authors:** Yage Zheng, Ling Xiao, Huiling Wang, Zhenhua Chen, Gaohua Wang

**Affiliations:** ^1^Mental health Center, Renmin Hospital of Wuhan University, Wuhan, China; ^2^Judicial Appraisal Institute, Renmin Hospital of Hubei Province, Wuhan, China

**Keywords:** non-suicidal self-injury (NSSI), retrospective research, detective rate, correlation factors, characteristics

## Abstract

**Background:**

Non-suicidal self-injury (NSSI) is an emerging public concern in both clinical and non-clinical settings, especially in the background of the coronavirus disease 2019 (COVID-19) pandemic. Nevertheless, knowledge of NSSI on a certain disease entity in the later stage of the pandemic was scarce.

**Objective:**

This study was conducted for the purpose of exploring the current occurrence and characteristics of NSSI in patients diagnosed with mood disorders (MDs) as well as its correlated factors in the later stage of the pandemic.

**Methods:**

Three hundred and forty-nine eligible subjects (M ± SD, 21.54 ± 7.62) admitted to a mental health center in Wuhan from 11 November 2021 to 31 January 2022 were included in our study. An umbrella questionnaire comprised of demographics, COVID-19-related factors, Yale-Brown Obsessive and Compulsive Scale (Y-BOCS), Pittsburgh Sleep Quality Index-Revised (PSQI-R), Mobile Phone Addiction Index (MPAI), and Ottawa Self-injury Inventory (OSI) was extended to each subject *via* shared QR code.

**Results:**

Of 349 patients with MDs included, 151 (43.27%) reported NSSI in the recent 1 month, among whom hand, lower arm/wrist, and scalp were the most hurt body parts, and cutting, hitting, and headbanging were the most adopted methods. “Own idea” was the most common origin of NSSI. In the logistic regression model, age bracket, family monthly income, occupation, level of obsessive-compulsive disorder (OCD) symptoms, sleep duration, withdrawal reaction to the mobile phone, and habits of using a mobile phone were independently associated with NSSI.

**Conclusion:**

It was revealed by our study that NSSI was quite prevalent among patients with MDs, especially among those students, adolescents, comorbid with OCD symptoms, inadequate sleeping hours, and suffering from withdrawal reaction to mobile phones. Further research on NSSI in various psychiatric disorders and even in non-clinical settings such as the community population was in urgent need since NSSI in China was not rare.

## Introduction

Non-suicidal self-injury (NSSI) is a series of deliberate actions a person takes to inflict certain damage on the human body. The self-destructive actions taken are diverse ranging from cutting, scratching, and overdrinking to eating or drinking things that are not edible, leading to varieties of damage in different categories, such as physical damage, chemical damage, and biological damage, to name only a few (1). NSSI exists in both clinical and non-clinical settings regardless of being diagnosed with or without psychiatric disorders (2). It was first reported in the 1980s, with a prevalence of 0.4%, and NSSI has been increasingly popular among the general population over time (3). It was suggested by Na Du's data from a single-centered study that the prevalence of NSSI among adolescent inpatients in the psychiatric department was increasing year by year over the past 5 years. Dramatically, this ratio was over 90% in 2020 and 2021 (4). Despite the early onset of NSSI engagement (usually between 12 and 16), people of all ages could be affected by this phenomenon. More than 5.1% of NSSI behaviors occurred for the first time during childhood. However, scant studies on NSSI among adults were extant, let alone among the elderly (3). Past experience showed a declining tendency in the prevalence of NSSI during adulthood (3), so we wondered if this finding also exists during coronavirus disease 2019 (COVID-19). It was reported that 4–6% of American adults have ever experienced at least one episode of NSSI in their lifetime, and this prevalence could be up to 10 or more times when considered in patients with various psychiatric disorders. While in the adolescent population with psychiatric disorders, over half of them might suffer from it (5). NSSI was believed to be the most common type of suicidal and self-harm behaviors in both lifetime and 12-month prevalence in a recent meta-analysis with 686,672 children and adolescents included (6). Unlike suicide, NSSI behaviors are not committed with the intention of ending one's own life. Nevertheless, an affinity between NSSI and suicide has been mentioned in previous studies. In a 12-year-follow-up longitudinal study, the occurrence of suicidal behaviors in patients with self-harm presented in the beginning of research was found to be 50 times as great as that in those without self-harm (7). For a person with either one situation of NSSI or suicidal attempt, lifetime co-occurrence of both of which could be ~70% (8). Meanwhile, the severity of obsessive-compulsive disorder (OCD) has usually been regarded as a prediction of suicide (9). Given the affinity between NSSI and suicide, it could be expected that an association between OCD and NSSI might exist. As far as I am concerned, scant evidence was shown on this point formerly. So, OCD in this study was another variable of interest. Also, sleep problems, such as poor sleep quality, sleep disturbances, and insufficient sleep duration, were believed to be connected to NSSI as per a meta-analysis (10). A revised version of PSQI containing 4 aspects was, therefore, adopted to check the tentative association with NSSI during COVID-19.

Originally esteemed as a symptom of borderline personality disorder or intentional self-injury with a sharp object in ICD-10, NSSI was not considered defined as a unique disease entity until the publication of DSM-5 (5). Still and all, ICD-10 is currently referred to as the diagnostic criterion for psychiatric disorders in most hospitals in China, and attention to screening and recognition of NSSI in clinical settings is thus largely neglected.

During the past 2 years, we human beings have suffered much from COVID-19, millions of infected patients lost their lives, and numerous residents were influenced both physically and mentally due to the virus *per se* as well as the socioeconomic impact arising from this. The popularity of online courses and the establishment of the home office model reshaped our way of life. In a large-scale study by Lan Guo, problematic Internet use was related to suicidal ideation or attempts (11), and we wondered if an association could be found between NSSI and mobile phone use likewise. According to an umbrella review by Xiong, the prevalence of depressive symptoms in the general population during the COVID-19 pandemic was between 14.6 and 48.3%, greater than what was estimated before the pandemic (12). Components of psychiatric disorders changed with different periods, and gone were the days when schizophrenia took the lead in the constituent ratio of various psychiatric disorders. As was shown in large cross-sectional research based on 32,552 Chinese individuals, the lifetime prevalence of psychiatric disorders went up from 1.27% in 1982 to 16.57% in 2013 (13). Although a relatively steady prevalence of schizophrenia was kept, MD, which ranked next to anxiety, is currently one of the most prevalent psychiatric disorders in China (13). Despite the fact that China had almost flattened the curve of COVID-19 since 29 April 2020, we are hardly kept informed of the situation of NSSI in the later stage of the pandemic. Quite limited amounts of studies were existing in relation to NSSI during the pandemic worldwide (14, 15). In China, only 2 studies explored the prevalence of NSSI during the pandemic, both of which showed high prevalence therein (4, 16). Nevertheless, both were targeted at adolescents and the peak of the pandemic, and no other age bracket was considered. Besides, information on NSSI in a certain psychiatric disorder is rare, especially in those high prevalent disease units in the clinical setting, for instance, MDs, which might not facilitate precision medicine. Given that MDs are quite prevalent in recent years, especially under the impact of COVID-19, it would be urgent to know more about NSSI behaviors among this population.

In this study, we first explored the occurrence of NSSI behaviors among patients with MD. Then, characteristics of NSSI behaviors among the sample population were introduced. Furthermore, we compared occurrences of NSSI behaviors in different groups of demographic factors, COVID-19-related factors, sleep status, and OCD levels, as well as phone dependence levels, ascertaining correlated factors with NSSI behaviors.

## Materials and methods

### Participants

From 11 November 2021 to 31 January 2022, inpatients were informed of accomplishing an established questionnaire on the first day they were admitted into the Mental Health Center of Renmin Hospital of Wuhan University.

Conditions of inclusion criteria were as follows: (1) young inpatients aged between 14 and 44 years, (2) with a principal diagnosis of MD in the outpatient department before being admitted, (3) without significant psychotic symptoms accompanied, and (4) those who had at least received elementary education.

**Figure 1:**
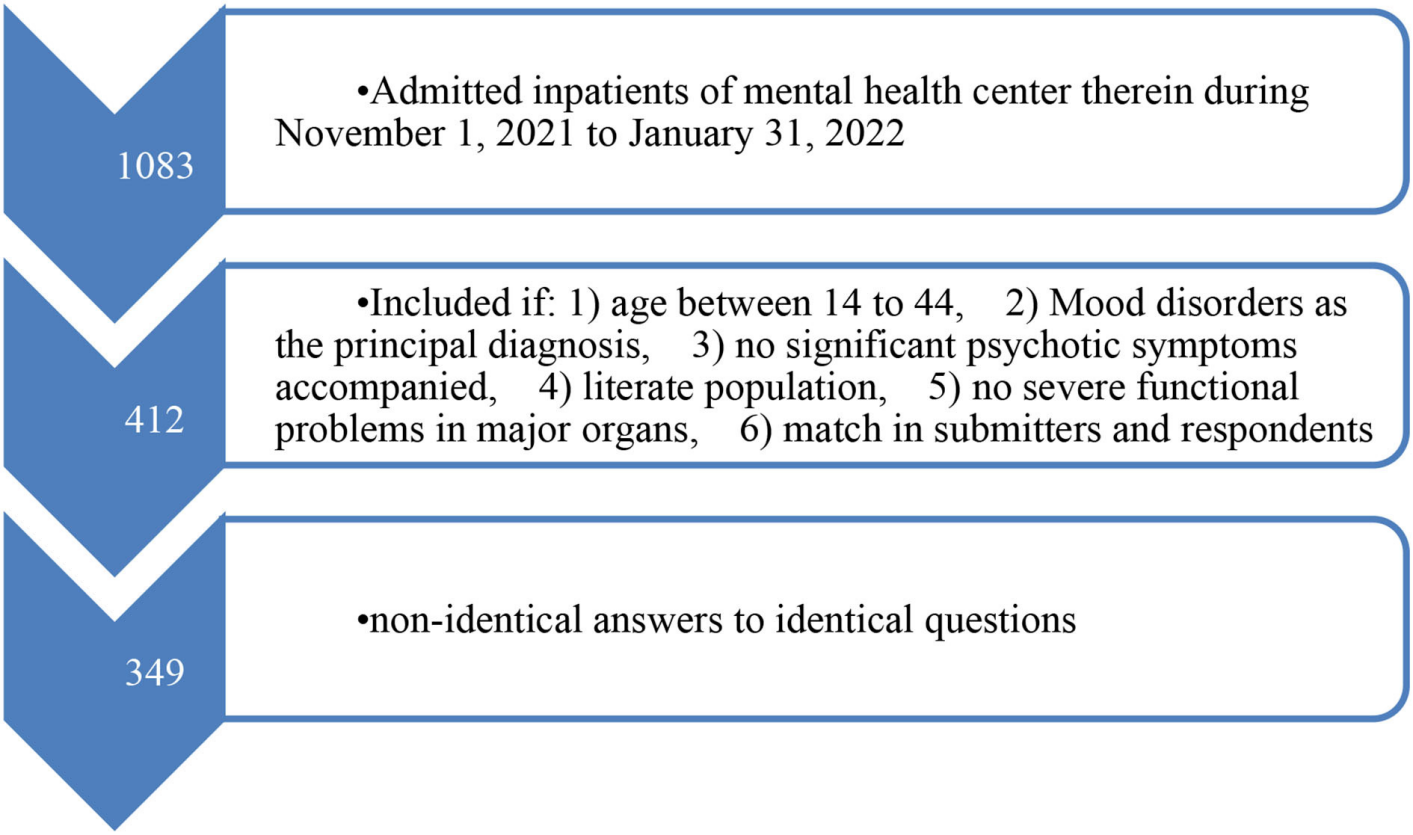
Flowchart.

Conditions of exclusion criteria were as follows: (1) aged <14 or above 45 years, (2) those who were primarily diagnosed with schizophrenia or had typical psychotic symptoms accompanied, (3) with severe physical problems in major organs such as heart, brain, and lung and (4) those who failed to accomplish the questionnaire on behalf of themselves (those who had other guys fill out the questionnaire instead). Diagnostic criteria of MD refer to ICD-10. The procedure of sample screening is shown in Figure 1.

All inpatients were voluntary to participate in filling out the questionnaire, and instruction has been extended to participants before they got started. Whenever and wherever those participants had a problem during the process, a trained psychiatrist was always available to address their issues. Meanwhile, participants could leave and quit at any time if they felt unpleasant. This research obtained oral ethical approval from Renmin Hospital of Wuhan University since no blood sample was drawn from the respondents for experimental purposes.

### Data collection

#### Type of questionnaire

To avoid frequent unnecessary contact between medical care workers and patients, Wenjuanxing, an online self-administered questionnaire network server was built as the vector.

#### Construction of questionnaire

The whole questionnaire could be divided into 6 parts as follows:

Items in part 1 consist of demographics, containing height, weight, age, sex, monthly family income, education level, employment status, career, home address, registered permanent residence, family structure, contact of second-hand smoking, initial age of touching electronic devices, possessions of electronic devices, and intensity of daily physical activities.

Part 2 reflected some factors associated with COVID-19, containing (1) whether your family members have ever participated in the war against COVID-19, (2) whether acquaintances around you have ever been infected with COVID-19, (3) to what extent your worries on engulfment of Wuhan City by COVID-19 would be, (4) subjective influence of COVID-19 personally, (5) total hours spent on the smartphone before and after the first outbreak of COVID-19, (6) working or studying hours spent on the smartphone before and after the first outbreak of COVID-19, and (7) distribution of hours spent on the smartphone during your leisure time. The Cronbach's α for this scale was 0.62.

Part 3 reflected levels of OCD symptoms with the Yale-Brown Obsessive and Compulsive Scale, a well-acknowledged scale for screening types and levels of obsessions and compulsions in different settings, especially in clinical settings (17, 18). Both validity and reliability of Y-BOCS have been examined in former studies in China, and despite originally being designed as a scale rated by the clinician, numerous studies have confirmed the availability and applicability of its self-administered version (19, 20). To begin with, participants should choose all their obsessions and compulsions in the questionnaire, and then they were faced with 10 questions to decide levels of their obsessive and compulsive symptoms according to the most disturbing ones. Each question consists of 5 choices, and the choice from top to bottom would be scored as “0” to “4,” respectively. The total point of these 10 questions would range from “0” to “40.” Respondents that scored <6 were deemed as having no significant OCD symptoms, and those who scored between “6” and “15” or “16” and “25” were deemed as having mild or moderate OCD symptoms, while those who scored up to “26” were deemed as having severe OCD symptoms (21). The Cronbach's α for this scale was 0.91 (22).

Part 4, to reflect the sleep status of subjects, PSQI-R (a revised version of PSQI) was adopted. In this simplified scale, we have 4 aspects of sleep under evaluation, namely, subjective sleep quality, early awakening, difficulty in falling asleep, and also, sleep duration. Each question had 4 choices and the choice from top to bottom was scored from “0” to “3,” respectively. The higher you scored in PSQI-R, the poorer your sleep status would be (23). The Cronbach's α for this scale was 0.71 (24).

In Part 5, we have the level of dependence on smartphones estimated among the respondents *via* the Mobile Phone Addiction Index (MPAI) scale. MPAI, a scale comprised of 17 questions, was classified into 4 dimensions as per different consequences arising from overusing mobile phones. Specifically, questions 1 to 7 represented “Inability to Control Craving,” questions 8 to 11 represented “Feeling Anxious & Lost,” questions 12 to 14 represented “Withdrawal or Escape,” and questions 15 to 17 represented “Productivity Loss.” Each question contained 5 choices, and the choice from “never” to “always” was scored as “1” to “5,” thereby the total score of which would range from “17” to “85.” The higher you scored, the more indulgent in mobile phone you turned out (25). The Cronbach's α for this scale was 0.91 (26).

As for the evaluation of non-suicidal self-injurious behaviors, we followed the definition described in Ottawa Self-Injury Inventory (OSI), in which NSSI meant a person did conduct some behaviors harmful to their body on purpose with any methods listed below: cutting, scratching, interfering with wound healing, bumping, hitting, and hair-pulling (27). Respondents were asked to decide if and how frequently NSSI occurred in the past 1, 6, 12 months and also, and 12 months ago. The Cronbach's α for this scale was 0.84 (28).

#### Quality control specification

To ensure a better quality of the questionnaire, we adopted 3 kinds of strategies. First, WeChat was the only designated app to complete the questionnaire, and respondents were asked to visit the web page of the questionnaire *via* scanning an established QR code. Second, considering the flexibility of personal scheduling, resuming from breakpoint was allowed, but notably, each respondent could submit no more than 1 questionnaire.

Finally, trap questions were introduced. Specifically, 2 pairs of identical questions were posed in a non-identical way, only if identical answers that were given could have confidence in the validity and authenticity of the questionnaire. For the link address of the electronic questionnaire, refer to https://www.wjx.cn/wjx/design/previewq.aspx?activity=135916788&s=1.

### Data analyses

Collected data were stored in excel data sheets (Microsoft, 2007). SPSS Statistics 24.0.0.0 (IBM, 2016) for Microsoft Windows 10, a 64-bit system, was applied as the tool for statistics. Categorical variables such as sex and education level were listed in the form of *n* (%), while continuous variables were listed in the form of mean ± SD. No clear boundaries were known between categorical and continuous variables for the contrived adjustment that would be made when considering our convenience. For example, “BMI,” an original metric variable, was listed as 4 classified levels from “underweight” to “obesity.”

**Figure 2:**
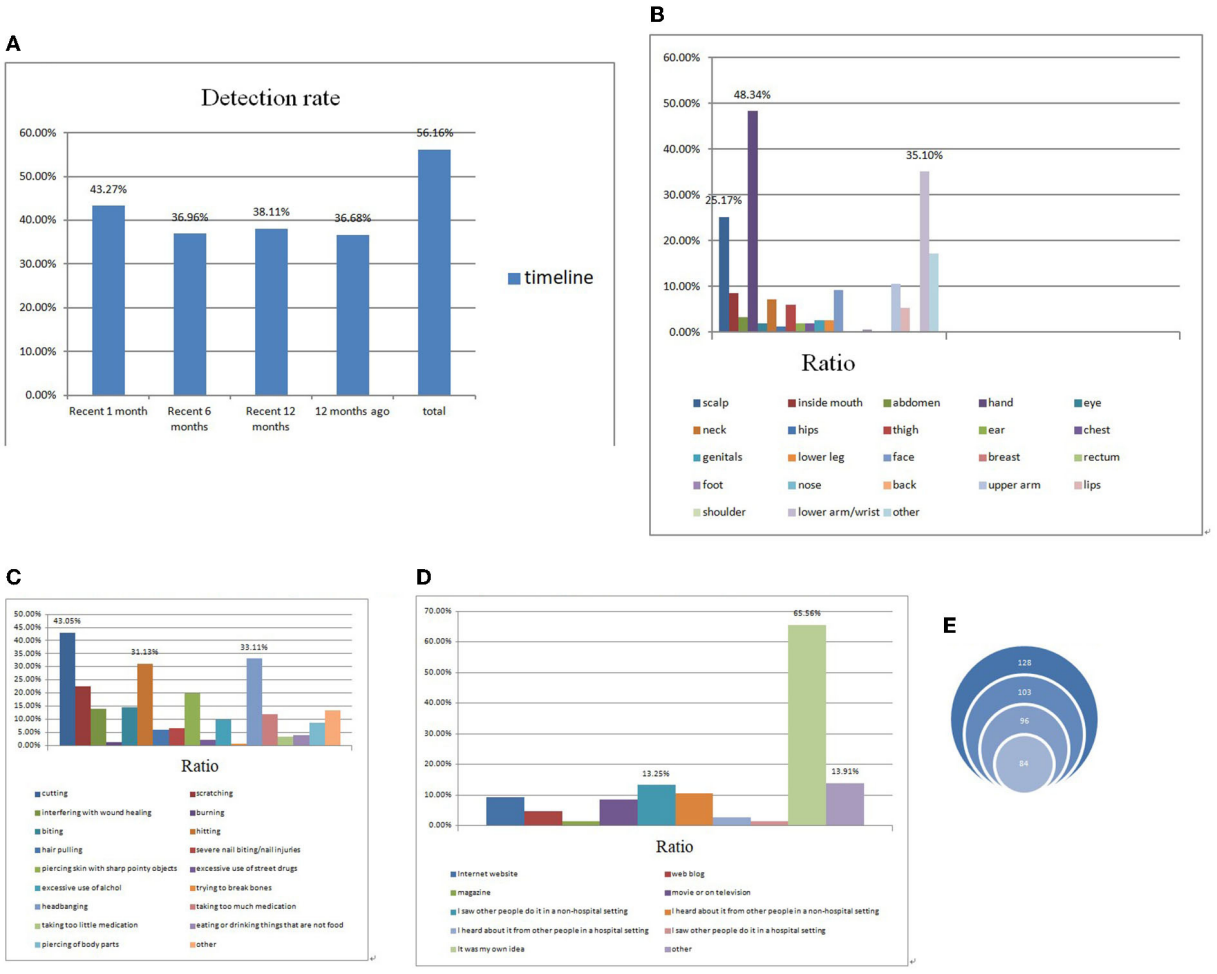
**(A)** Detection rate of non-suicidal self-injury (NSSI) among selected subjects. **(B)** Most hurt areas of NSSI among selected subjects. **(C)** Most adopted methods of NSSI among selected subjects. **(D)** Origins of NSSI idea among selected subjects. **(E)** Repetitive NSSI behaviors among selected subjects.

First, descriptive statistics of data was obtained (Table 1, Figures 2A–E). In our study, whether a subject engaged in NSSI currently (in recent 1 month) was esteemed as the dependent variable (categorical), while independent variables consist of both categorical and continuous ones. Then, a preliminary screening of associated variables with NSSI behaviors was conducted *via* the chi-square test for categorical independent variables and *via* Student's *t*-test/Mann–Whitney–Wilcoxon test for continuous independent ones (Supplementary Table 1). With that, all the screened variables with significance above were included in a binary logistic regression model with a screening method of forward: LR, and thereby the associated factors with NSSI behaviors came out (Table 2). *P* <0.05 was considered statistically significant.

**Table 1:** Socio-demographics, pandemic-related factors, obsessive compulsive disorder (OCD) symptoms, sleep quality, and phone dependence of subjects.

**Variable**		**Sample, *n* (%)**	**Mean ±SD**
BMI	Underweight	77 (22.06)	
	Normal	183 (52.44)	
	Overweight	56 (16.05)	
	Obesity	33 (9.45)	
Age bracket	Adolescent	132 (37.82)	
	Young adults	217 (62.18)	
Sex	Male	156 (44.70)	
	Female	193 (55.30)	
Monthly family income (RMB)	<3 K	38 (10.89)	
	3–5 K	100 (28.65)	
	5–10 K	125 (35.82)	
	>10 K	86 (24.64)	
Education level	Junior high school	74 (21.20)	
	Senior high school	117 (33.52)	
	Junior college	44 (12.61)	
	Bachelor degree or above	114 (32.66)	
Present situation	Employed or in school	265 (75.93)	
	Unemployed or dropout	84 (24.07)	
Occupation	Students	231 (66.19)	
	Others	118 (33.81)	
Usual place of residence	Wuhan	101 (28.94)	
	Other places	248 (71.06)	
Hometown	Urban	117 (33.52)	
	Town	90 (25.79)	
	Rural	142 (40.69)	
Structure of family	Nuclear family	196 (56.16)	
	Extended family	94 (26.93)	
	Single parent or blended family	59 (16.91)	
Passive smoking	Never	234 (67.05)	
	1 day a week	27 (7.74)	
	2 to 3 days a week	21 (6.02)	
	Over 3 days a week	19 (5.44)	
	Almost every day	48 (13.75)	
Initial age of touching electronic devices	Preschool	22 (6.30)	
	School age	157 (44.99)	
	Adolescent	126 (36.10)	
	Young adults	44 (12.61)	
Possessions of electronic devices	Smartphone	101 (28.94)	
	Smartphone and other devices	232 (66.48)	
	No Smartphone	16 (4.58)	
Intensities of physical activities	Light	130 (37.25)	
	Moderate	74 (21.20)	
	Heavy	145 (41.55)	
Relatives as volunteers in the combat against COVID-19	No	270 (77.36)	
	Yes	79 (22.64)	
Acquaintances infected with COVID-19	No	323 (92.55)	
	Yes	26 (7.45)	
Worries on re-occurrence of COVID-19 in a large scale	Never	130 (37.25)	
	Somewhat	147 (42.12)	
	Quite a bit	48 (13.75)	
	Always	24 (6.88)	
Influenced by COVID-19	No	104 (29.80)	
	Yes	245 (70.20)	
Time spent on Smartphone before/after COVID-19	<1 h	44 (12.61)/9 (2.58)	
	1–3 h	93 (26.65)/35 (10.03)	
	3–5 h	95 (27.22)/79 (22.64)	
	5–7 h	52 (14.90)/96 (27.51)	
	>7 h	65 (18.62)/130 (37.25)	
Time spent on work and study with Smartphone before/after COVID-19	<1 h	98 (28.08)/35 (10.03)	
	1–3 h	109 (31.23)/75 (21.49)	
	3–5 h	66 (18.91)/75 (21.49)	
	5–7 h	39 (11.17)/72 (20.63)	
	>7 h	37 (10.06)/92 (26.36)	
Type of amusement with Smartphone at leisure time before/after COVID-19	Chat	62 (17.77)/50 (14.33)	
	Video	76 (21.78)/82 (23.50)	
	Music	65 (18.62)/55 (15.76)	
	Games	82 (23.50)/66 (18.91)	
	Work and study	39 (11.17)/64 (18.34)	
	Other	25 (7.16)/32 (9.17)	
OCD symptoms	No	94 (26.93)	
	Mild	125 (35.82)	
	Moderate	97 (27.79)	
	Severe	33 (9.46)	
PSQI-R			10.21 ± 3.30
Subjective feeling			2.60 ± 0.92
Early awakening			2.86 ± 1.10
Difficulty in falling asleep			2.74 ± 1.19
Sleep duration			2.00 ± 1.06
MPAI			37.81 ± 12.84
Feeling Anxious & Lost			8.89 ± 3.95
Inability to Control Craving			16.91 ± 6.49
Productivity Loss			4.16 ± 1.96
Withdrawal or Escape			7.84 ± 2.94

**Table 2:** Variables associated with non-suicidal self-injury (NSSI) behaviors in the model of binary logistic regression.

**Variable**	**B**	**OR**	**95% CI**
Age bracket			
Adolescent		1	Ref
Young adult	−0.683	0.505^*^	(0.278, 0.918)
Family monthly income			
<3 K		1	Ref
3–5 K	0.446	1.561	(0.635, 3.842)
5–10 K	−0.486	0.615	(0.252, 1.501)
>10 K	1.047	2.850^*^	(1.116, 7.278)
Occupation			
Students		1	Ref
Others	−0.684	0.505^*^	(0.259, 0.982)
Type of amusement with Smartphone at leisure time after COVID-19			
Chat		1	Ref
Video	−0.861	0.423^*^	(0.187, 0.956)
Music	−0.346	0.707	(0.304, 1.646)
Games	−1.119	0.327^**^	(0.145, 0.736)
Work and study	−1.899	0.150^***^	(0.048, 0.468)
Other	−1.348	0.260^*^	(0.077, 0.881)
OCD symptoms			
No		1	Ref
Mild	0.682	1.977	(0.968, 4.039)
Moderate	1.338	3.810^***^	(1.747, 8.311)
Severe	1.684	5.388^**^	(1.863, 15.581)
Sleep duration	0.399	1.491^**^	(1.152, 1.928)
Feeling anxious and lost	0.127	1.135^***^	(1.056, 1.221)

*p <0.05, **p < 0.01, ***p < 0.001.

## Results

Totally, 1,083 questionnaires were extended, among which 412 were collected. As per the criteria, 349 eligible subjects with a mean age of 21.54 ± 7.62 years were included, of whom 37.82% were in the age bracket of adolescent (aged below 18 years), and 44.70% accounted for the male subjects. As for subtypes of MD, 248 were depressive episodes, 33 were bipolar disorder, 47 were maniac episodes, 11 were recurrent depression, and 10 were dysthymia/cyclothymia. Detailed information about demographics and other necessary factors of the sample are introduced in Table 1.

Of all the subjects (*n* = 349), 151 (43.27%) reported engaging in NSSI in the recent 1 month, and the detection rate of NSSI among whom in the past 6, 12, and 12 months ago was 36.96, 38.11, and 36.68%, respectively.

“Hand” is the most targeted body part of being hurt during the process of NSSI with a proportion of 46.94%, followed by “lower arm or wrist” with a proportion of 32.14%, and then, “upper arm or elbow” of 13.27%.

Talking about methods of NSSI behaviors, nearly half of the subjects with NSSI (48.47%) had ever tried such self-injury behaviors by cutting themselves. Compared with those hurt by headbanging, the proportion of subjects who engaged in NSSI by means of hitting was 28.06%. Around a quarter adopted methods of scratching during NSSI.

Of all 196 subjects who have ever engaged in NSSI, the majority (64.80%) reported coming up with the idea of NSSI out of their own thoughts. While 14.80% attributed the idea of NSSI to “I saw other people do it in a non-hospital setting,” 15.31% reported other origins of the idea.

Of all 128 subjects who had ever engaged in NSSI 12 months ago, the bulk (80.47, 75, and 65.63%, respectively) reported that they still tried out NSSI in the past 12, 6, and 1 month. All characteristics of NSSI among the subjects are summarized in Figures 2A–E.

Refer to Supplementary Table 1 for all possible associated factors with NSSI. As shown in Table 2, variables of age bracket, family monthly income, occupation, family structure, intensities of physical activities, OCD symptoms, sleep duration, MPAI scores, and inability to control craving were associated with NSSI. The occurrence of NSSI in young adults was as 0.445-fold as that in adolescents (*p* < 0.05). The occurrence of NSSI in subjects whose family monthly income was up to 10,000 was as 6.249-fold as that in those with family monthly income below 3,000 (*p* < 0.001). The occurrence of NSSI among non-student subjects was as 0.420-fold as that among students (*p* < 0.05). The occurrence of NSSI among subjects with extended family, single parent, or blended family was, respectively, as 2.219-fold (*p* < 0.05) and 6.249-fold (*p* < 0.001) as that among those with the nuclear family. The occurrence of NSSI in subjects with moderate physical activities was as 0.329-fold as that in light ones (*p* < 0.01). The occurrence of NSSI in subjects with a mild level of OCD symptoms was as 2.752-fold as that in those without OCD symptoms (*p* < 0.01), while the fold in those with a moderate and severe level of OCD symptoms was 5.239 (*p* < 0.001) and 4.645 (*p* < 0.05), respectively. Each increased level of sleep duration would increase the occurrence of NSSI by 65.5% (*p* < 0.001). Each increased MPAI score would increase the occurrence of NSSI by 12.7% (*p* < 0.001). Each increased score of inability to control craving would decrease the occurrence of NSSI by 10.8% (*p* < 0.05). As to the type of amusement with smartphones in leisure time before COVID-19, the occurrence of NSSI among subjects who mainly spent time on video, music, games, work, and study was as 0.423-, 0.707-, 0.327-, 0.150-fold, respectively, as that in those who spent time mainly on chatting.

## Discussion

After several rounds of mutation from the delta, lambda to Omicron, the COVID-19 virus seemed tougher to cope with. According to the data from the American Academy of Pediatrics and the Children's Hospital Association, amounts of child COVID-19 cases spiked dramatically in January with over 1.1 million were reported in 1 week, nearly five times the rate of that at the peak of last winter's surge (29). While I was writing this manuscript, a new wave of the pandemic has also landed in China once again. In the past week by the end of 6 March, 302.173 thousand COVID-19 cases were found, posing a tremendous threat to the public, especially to those with psychological problems. In our study, the target population was patients with MD aged 14–44 years, and as was shown within, 43.27% of subjects (58.33% in adolescents and 34.10% in young adults) reported NSSI currently, indicating that these phenomena were not rare among this population, even in the remission stage of COVID-19. In our study, the scalp, hand, lower arm, and wrist were the most chosen body parts for being hurt, which went in parallel with adopted methods of NSSI, namely, cutting, hitting, and headbanging. Very few studies in China talked about the methods of NSSI, but agreement on frequent methods of cutting and hitting seemed to be true, and the difference in methods of NSSI might be due to the difference in the selected population (30). Understandably, body parts such as the scalp, hand, and wrist were often exposed without cover, and once the self-harm idea came to a person's mind, these body parts were surely at risk. Usually covered by clothing, the lower arm might be hurt for its convenience and stealthiness, and a person could switch swiftly from a state of hurting himself/herself on pushing up sleeves to another state of concealing any scratch on pushing down sleeves without being detected. Similar to previous studies, the origins of self-injury ideas were diverse, including but not limited to both auditory and visual self-harm information from videos, movies, images, TV shows, Internet websites, and anecdotes in the real world. As for how these behaviors came out, social contagion might be an important mediating factor. *Via* frequent contact with peer pressure, cyber-bullying, glamorized images/videos of the self-injury process, abetment and implication of self-injury online or offline, adolescents, and young adults, especially those vulnerable subjects, often came to terms with self-injury (31). During psychological counseling in the ward, we found quite a few adolescent patients that were members of illegal WeChat/QQ groups, in which feelings, experiences, and methods of self-injury were often shared, despite no specific data being acquired, which was sheer alarming for nobody knows what would happen once this situation went spreading around out of control. We do not hope this phenomenon be the next blue whale challenge. Dopamine, which is hailed as a happiness hormone, often decreased in level among patients with MD, especially among those with a depressive episode (32, 33). NSSI, as a kind of acute stimulating behavior, could upregulate the level of dopamine in our body, which in turn caused further addiction to it (34), that was why so many patients came up with the idea of NSSI, and once they tried, they were inclined to try again.

In our study, the detection rate of NSSI in adolescents was 1.98 times more than that in young adults (*p* < 0.05), although both of which were relatively great. That was consistent with Nock's observation showing that early adolescence was the most common age of NSSI onset, while NSSI was sometimes replaced by other kinds of dysfunctional behaviors in late adolescence (35, 36). Paradoxical findings were found with regard to the prevalence of NSSI in men or women, which might be attributed to heterogeneity in methodology, sample population, definition of NSSI, and so on (30). Notably, the age of male subjects in our study was similar to that of female ones, and still, no significant difference in the detection rate of NSSI was found between sexes, which meant that NSSI was prevalent regardless of sexes in patients with MD. To our surprise, those patients with better economical conditions seemed more likely to try NSSI, say, patients with MD with family monthly income over 10,000 were 2.850 times the risk of trying NSSI than those whose income was below 3,000. As we know, higher income meant more time spent on the job, which was often at the sacrifice of gathering and emotional communication with family members. Young people who were at the age of character-shaping were in great desperate need of emotional support, especially for patients with a mental disorder, whose recognition, emotional regulation functions, and coping skills were largely undermined (37), and proper and timely guidance from family members was of great importance. Interpersonal and peer identification, peer victimization and bullying, and peer acceptance and rejection could exert tremendous impacts on NSSI thoughts and behaviors (10). A high occurrence of NSSI in students also called for more attention from the education system. Both cross-sectional and longitudinal studies had presented an idea of the potential association between poor sleep and higher risk of NSSI (10). As was reported, future NSSI among adolescents could be predicted by sleep problems several years ago, especially by sleep duration. According to Fang's observation, the non-clinical adolescent with inadequate sleeping hours was at up to 5 times the risk of trying NSSI compared with those with adequate sleep (10). Likewise, sleep duration also stands out as an independently associated factor with NSSI, and each decreased level of sleep duration would increase the risk of NSSI by 49.1% (*p* < 0.01). Besides, we found that phone dependence was closely associated with NSSI. Patients who felt more anxious and lost without a phone were more likely to try NSSI, specifically, each increased score in the dimension of withdrawal reaction would put patients with MD at a higher risk of NSSI by 13.5% (*p* < 0.001). Mobile phones *per se* might not trigger NSSI directly; however, mood instability and emotional disturbances that followed would drive vulnerable subjects crazy, impulse built, and built within and finally, and NSSI was applied as a relief. That was quite similar to other kinds of pathological uses such as Internet addiction (38), alcohol dependence (39), and tobacco dependence (40). Our study showed that OCD symptoms were extremely common among patients diagnosed with MD, and a positive correlation was found between the level of OCD symptoms and the risk of NSSI. To a certain extent, some subtypes of OCD symptoms were originally self-harm thoughts and behaviors, for example, aggressive obsession, in which a person felt the impetus to hurt himself or others, washing/cleaning, compulsion, in which a person kept washing till erosion of skin, or trichotillomania, in which a person could not help pulling hairs (41, 42). In addition, the association between NSSI and psychiatric comorbidities had been mentioned in depressive disorder and OCD symptoms (43). Additionally, the type of amusement with smartphones in leisure time before COVID-19 was concerned with NSSI. Compared with those who preferred chatting through smartphones, patients who mainly leaned on video, music, games, and even other types, of amusement were less likely to engage in NSSI. The explanation for this phenomenon might be targeted in dual aspects. First, the effect of social contagion, in which self-harm feelings or experiences could be spread out *via* chatting or communicating online, has been always esteemed as a risk factor for NSSI (31). Second, demands for amusement such as games or music, once met, might improve life satisfaction levels, thus reducing the possibility of NSSI (44). Meanwhile, occupation of time in a relatively light-hearted way might reduce an individual's chances of being exposed to NSSI. Nevertheless, scarcely did it mean the strategy of advocating more music or games in using smartphones or less chatting with others. Anyhow, it reminds us of the necessity of improving the quality of chatting as well as keeping a good blend of distribution of time on smartphones. Future studies could further investigate these tentative findings.

## Limitations and prospects

Several limitations have to be presented therein. In the first place, suspension of extra bed service and establishment of the buffer zone in the ward due to COVID-19 greatly pare the admission rate of psychiatric patients, making it hard to acquire a considerable size of sample in a limited span. Second, the nature of a cross-sectional study lacks convincible evidence and confidence in the establishment of causality between associated factors and NSSI. With these in mind, we are plotting to enlarge the sample volume in time when necessary, and simultaneously, follow-up research on the existing sample is also under consideration since the re-outbreak of the pandemic domestically is underway. Then, a multicenter study combining data from different areas and different clinical or non-clinical settings in China in the future would provide us with a comprehensive understanding of NSSI. Fourth, information on NSSI of children and the elderly was not included since the targeted age in our study was 14–44 years, and future study on population with a more wide-ranging age bracket is essential. Finally, bound by sample size, many interesting problems such as the correlation between NSSI methods and type of OCD symptoms, discrepancies of NSSI in patients with different types of MD, and the influence of antidepressants on NSSI were not concerned, and we are expecting more related studies in the future.

## Conclusion

Our study provided us with timely information on the current occurrence and characteristics of NSSI among patients with MD in the later stage of COVID-19, showing a great prevalence of NSSI among this population. Besides, several associated factors with NSSI were reported. Given the volatile and unpredictable situation of COVID-19 domestically and abroad, more attention should be paid to screening and detection of NSSI among the general population, especially among those at high risk.

## Data availability statement

The raw data supporting the conclusions of this article will be made available by the authors, without undue reservation.

## Ethics statement

The studies involving human participants were reviewed and approved by the Ethics Committee of Renmin Hospital of Wuhan University. Written informed consent to participate in this study was provided by the participants' legal guardian/next of kin.

## Author contributions

Conceptualization and design: YZ and LX. Methodology: YZ, LX, and ZC. Propaganda and mobilization, software operation, and statistics: YZ and ZC. Data collection, cleansing, and original draft paper writing: YZ. Supervision and verification: ZC, HW, and GW. Review and editing: LX and GW. Project administration and funding acquisition: GW. All authors contributed to the article and approved the submitted version.

## Funding

This work was supported by the National Natural Science Foundation of China: 81871072 and 82071523, the Tengfei Program of Wuhan University: TFLC2018001, and Key Research and Development Program of Hubei Province: 2020BCA064.

## Conflict of interest

The authors declare that the research was conducted in the absence of any commercial or financial relationships that could be construed as a potential conflict of interest.

## Publisher's note

All claims expressed in this article are solely those of the authors and do not necessarily represent those of their affiliated organizations, or those of the publisher, the editors and the reviewers. Any product that may be evaluated in this article, or claim that may be made by its manufacturer, is not guaranteed or endorsed by the publisher.

## Abbreviations

NSSI: non-suicidal self-injury
COVID-19: coronavirus disease 2019
MD: mood disorders
YBOCS: Yale-Brown Obsessive and Compulsive Scale
PSQI-R: Pittsburgh Sleep Quality Index-Revised
MPAI: Mobile Phone Addiction Index
OSI: Ottawa Self-Injury Inventory
QR code: quick response code
OCD: obsessive-compulsive disorder
ICD-10: The International Statistical Classification of Diseases and Related Health Problems 10th Revision
DSM-5: Diagnostic and Statistical Manual of Mental Disorders 5th Revision
BMI: body mass index.
