# Supplementary material for: A retrospective research on non-suicidal self-injurious behaviors among young patients diagnosed with mood disorders
Source: Front Psychiatry. 2022 Jul 22;13:895892. doi: 10.3389/fpsyt.2022.895892 (PMC9354581; doi:10.3389/fpsyt.2022.895892)
Supplement: Supplementary file 1 [file Table_1.DOCX]

Supplementary Table 1, Distribution of NSSI behaviors among the sample population

| Variable | NSSI behaviors | |  |  |
| --- | --- | --- | --- | --- |
|  | No(n=198) | Yes(n=151) | χ^2^ | P |
| BMI |  |  |  |  |
| Underweight | 44 | 33 | 3.126 | 0.373 |
| Normal | 97 | 86 |  |  |
| Overweight | 35 | 21 |  |  |
| Obesity | 22 | 11 |  |  |
| Age bracket |  |  |  |  |
| Adolescent | 55 | 77 | 19.633 | 0.000 |
| Youth | 143 | 74 |  |  |
| Sex |  |  |  |  |
| Male | 108 | 48 | 17.949 | 0.000 |
| Female | 90 | 103 |  |  |
| Monthly family income | | | | |
| ＜3K | 24 | 14 | 13.371 | 0.004 |
| 3-5K | 53 | 47 |  |  |
| 5-10K | 84 | 41 |  |  |
| ≥10K | 37 | 49 |  |  |
| Education level | | | | |
| Junior high school | 36 | 38 | 6.886 | 0.076 |
| Senior high school | 61 | 56 |  |  |
| Junior college | 26 | 18 |  |  |
| Bachelor degree or above | 75 | 39 |  |  |
| Present situation | | | | |
| Employed or in school | 141 | 124 | 5.576 | 0.018 |
| Unemployed or dropout | 57 | 27 |  |  |
| Occupation |  |  |  |  |
| Students | 112 | 119 | 18.938 | 0.000 |
| Others | 86 | 32 |  |  |
| Usual place of residence | | | | |
| Wuhan | 62 | 39 | 1.253 | 0.263 |
| Other place | 136 | 112 |  |  |
| Hometown |  |  |  |  |
| Urban | 60 | 57 | 4.396 | 0.111 |
| Town | 48 | 42 |  |  |
| Rural | 90 | 52 |  |  |

| Structure of family | | | | |
| --- | --- | --- | --- | --- |
| Nuclear family | 121 | 75 | 8.198 | 0.017 |
| Extended family | 53 | 41 |  |  |
| Single parent or blended family | 24 | 35 |  |  |

| Passive smoking |
| --- |

| No | 137 | 97 | 0.951 | 0.329 |
| --- | --- | --- | --- | --- |
| Yes | 61 | 54 |  |  |

| Initial age of touching electronic devices |
| --- |

| Preschool | 15 | 7 | 17.600 | 0.001 |
| --- | --- | --- | --- | --- |
| School age | 75 | 82 |  |  |
| Adolescent | 72 | 54 |  |  |
| Youth | 36 | 8 |  |  |

| Possessions of electronic devices |
| --- |

| Smartphone | 63 | 38 | 5.589 | 0.061 |
| --- | --- | --- | --- | --- |
| Smartphone and other devices | 130 | 102 |  |  |
| No Smartphone | 5 | 11 |  |  |

| Intensities of physical activities |
| --- |

| Light | 77 | 53 | 1.934 | 0.380 |
| --- | --- | --- | --- | --- |
| Moderate | 45 | 29 |  |  |
| heavy | 76 | 69 |  |  |

| Relatives as volunteers in the combat against COVID-19 |
| --- |

| No | 155 | 115 | 0.221 | 0.639 |
| --- | --- | --- | --- | --- |
| Yes | 43 | 36 |  |  |

| Acquaintances infected with COVID-19 |
| --- |

| No | 185 | 138 | 0.519 | 0.471 |
| --- | --- | --- | --- | --- |
| Yes | 13 | 13 |  |  |

| Worries on re-occurrence of COVID-19 in a large scale |
| --- |

| Never | 80 | 50 | 9.487 | 0.023 |
| --- | --- | --- | --- | --- |
| Somewhat | 88 | 59 |  |  |
| Quite a bit | 22 | 26 |  |  |
| Always | 8 | 16 |  |  |

| Influenced by COVID-19 |
| --- |

| No | 74 | 30 | 12.550 | 0.000 |
| --- | --- | --- | --- | --- |
| Yes | 124 | 121 |  |  |

| Time spent on Smartphone before COVID-19 |
| --- |

| ＜1 | 22 | 22 | 3.162 | 0.531 |
| --- | --- | --- | --- | --- |
| 1-3 | 49 | 44 |  |  |
| 3-5 | 58 | 37 |  |  |
| 5-7 | 33 | 19 |  |  |
| ＞7 | 36 | 29 |  |  |

| Time spent on Smartphone after COVID-19 |
| --- |

| ＜1 | 7 | 2 | 8.952 | 0.062 |
| --- | --- | --- | --- | --- |
| 1-3 | 24 | 11 |  |  |
| 3-5 | 49 | 30 |  |  |
| 5-7 | 56 | 40 |  |  |
| ＞7 | 62 | 68 |  |  |

| Time spent on work and study with Smartphone before COVID-19 |
| --- |

| ＜1 | 43 | 55 | 12.731 | 0.013 |
| --- | --- | --- | --- | --- |
| 1-3 | 63 | 46 |  |  |
| 3-5 | 47 | 19 |  |  |
| 5-7 | 22 | 17 |  |  |
| ＞7 | 23 | 14 |  |  |

| Time spent on work and study with Smartphone after COVID-19 |
| --- |

| ＜1 | 19 | 16 | 10.239 | 0.037 |
| --- | --- | --- | --- | --- |
| 1-3 | 52 | 23 |  |  |
| 3-5 | 47 | 28 |  |  |
| 5-7 | 35 | 37 |  |  |
| ＞7 | 45 | 47 |  |  |

| Type of amusement with Smartphone at leisure time before COVID-19 |
| --- |

| Chat | 21 | 41 | 27.959 | 0.000 |
| --- | --- | --- | --- | --- |
| Video | 48 | 28 |  |  |
| Music | 30 | 35 |  |  |
| Games | 51 | 31 |  |  |
| Work and study | 31 | 8 |  |  |

| Other | 17 | 8 |  |  |
| --- | --- | --- | --- | --- |
| Type of amusement with Smartphone at leisure time after COVID-19 | | | | |
| Chat | 19 | 31 | 17.733 | 0.003 |
| Video | 55 | 27 |  |  |
| Music | 24 | 31 |  |  |
| Games | 42 | 24 |  |  |
| Work and study | 36 | 28 |  |  |
| Other | 22 | 10 |  |  |
| OCD symptoms | | | | |
| No | 74 | 20 | 39.246 | 0.000 |
| Mild | 75 | 50 |  |  |
| Moderate | 39 | 58 |  |  |
| Severe | 10 | 23 |  |  |
|  | Mean±SD |  | Z | P |
| PSQI-R | 9.60±3.46 | 11.01±2.90 | -3.952 | 0.000 |
| Subjective feeling | 2.50±0.94 | 2.74±0.87 | -2.581 | 0.010 |
| Early awakening | 2.72±1.14 | 3.05±1.02 | -2.716 | 0.007 |
| Difficulty in falling asleep | 2.57±1.22 | 2.97±1.10 | -3.110 | 0.002 |
| Sleep duration | 1.81±1.03 | 2.25±1.04 | -4.141 | 0.000 |
| MAPI | 34..44±12.42 | 42.21±12.06 | -5.715 | 0.000 |
| Feeling Anxious & Lost | 7.77±3.43 | 10.37±4.11 | -6.123 | 0.000 |
| Inability to Control Craving | 15.56±6.38 | 18.69±6.21 | -6.410 | 0.000 |
| Productivity Loss | 3.80±1.87 | 4.64±1.99 | -4.654 | 0.000 |
| Withdrawal/  Escape | 7.32±2.92 | 8.52±2.83 | -3.797 | 0.000 |
